# Linking Decent Work and Well-Being Among Chinese Millennial Employees: A Psychology of Working Perspective

**DOI:** 10.3389/fpsyg.2022.909423

**Published:** 2022-05-26

**Authors:** Wei Wan, Tingting Cao

**Affiliations:** School of Business Administration, Jiangxi University of Finance and Economics, Nanchang, China

**Keywords:** millennial employees, decent work, well-being, psychology of working theory, need satisfaction

## Abstract

Drawing from the psychology of working theory, this study aims to understand how decent work is related to employee well-being. Specifically, it explored the role of need satisfaction (i.e., survival, social contribution, and self-determination) in the relationship between decent work and employee well-being, and compared the mediating effects of the three types of need satisfaction. After collecting a sample of 421 millennial employees in China through online questionnaires, the study conducted the analysis of the data and found that decent work positively predicted well-being of millennial employees. While social contribution need satisfaction and self-determination need satisfaction partially mediated the effect of decent work on well-being of millennial employees, the mediating effect of survival need satisfaction was not significant. Compared with social contribution need satisfaction, self-determination need satisfaction had a more significant mediating effect on well-being of millennial employees. The study does extend the literature on the antecedents of employee well-being and the results can offer some implications for managers to enhance well-being of millennial employees.

## Introduction

In the context of China’s social reform and economic transformation, employees’ values, attitudes and behaviors have undergone many changes. In particular, the large-scale entry of millennial employees into the workplace has reshaped the talent characteristics of the labor market. Millennial employees, known as the “Y generation” in the west (Hansford, 2002), refer to workers born in the 1980s and 1990s in China (Fang et al., 2020). Under the influence of one-child family policy (i.e., each family can only have one child) implemented since 1980, Chinese millennials have received a great deal of attention from their family (Zhao, 2018). As a result, they tend to be self-centered, and care much about their own inner feelings and psychological needs (Warner and Zhu, 2018). Out of the need for work-and-life balance (Twenge, 2010), millennial employees not only desire to obtain well-being through their work, but also hope to have plenty of time to enjoy their life (Lee et al., 2017). Well-Being can reflect employees’ satisfaction with their work and life, and exert a certain influence on employees’ work attitude and behavior (Amah, 2009). Existing studies have shown that well-being can not only improve employees’ loyalty to organizations (Milyavskaya and Koestner, 2011), but also contribute to employees’ job performance (Gillet et al., 2012). Therefore, it is of great theoretical and practical significance to study how to improve well-being of millennial employees.

The psychology of working theory (PWT) proposes that decent work can improve individuals’ well-being by satisfying their needs for survival, social contribution and self-determination (Duffy et al., 2016). Empirical studies have also proved that decent work can predict job and life satisfaction as well as physical health (Buyukgoze-Kavas and Autin, 2019; Duffy et al., 2019), and need satisfaction *via* decent work has been shown to significantly mediate these relationships (Duffy et al., 2019). Although well-being has been investigated by many scholars, there has been minimal research on this subject for Chinese millennial workforce. In addition, it remains unknown whether there are differences in the mediating effects of different types of need satisfaction in the relationship between decent work and well-being of millennial employees.

Based on PWT, this study address these gaps by discussing the internal mechanism through which decent work influences well-being of Chinese millennial employees, and comparing the mediating effects of satisfaction of survival needs, social contribution needs and self-determination needs. It is hoped that the results of this study will elaborate whether and how need satisfaction plays a role in the link between decent work and well-being of millennial employees.

## Theoretical Framework and Hypotheses Development

### Psychology of Working Theory

Vocational psychologists have pointed out that work plays an essential role in people’s lives (Fouad, 2007) since it is closely related to one’s self-concept, and the experience of work is associated with one’s sense of self-esteem, sense of control, coherence of self-identity, and physical and psychological well-being (Blustein et al., 2008). Decent work was first conceptualized at the macro level “to promote opportunities for men and women to obtain decent and productive work on the premise of freedom, equality, security, and human dignity” (International Labor Organization, 1999 p. 3). Later, it was adopted into PWT as a core concept which is defined as “(1) work that offers physical and interpersonal safe working conditions, (2) hours that allow for free time and rest, (3) organizational values that complement one’s family and social values, (4) adequate compensation, and (5) access to adequate healthcare” (Duffy et al., 2016, p. 130).

According to PWT, decent work can enable individuals to obtain well-being *via* meeting basic human needs which can be divided into survival needs, social contribution needs, and self-determination needs involving autonomy, competence, and relatedness (Duffy et al., 2016). The final set of needs can also be found in the self-determination theory (SDT) which emphasizes how workplace contexts or individual differences affect one’s work attitudes, behaviors, health, and well-being through psychological needs (Deci and Ryan, 2008). Nevertheless, the main differences between these two theories lie in PWT’s focus on decent work as the primary antecedent variable of need satisfaction and the integration of survival needs and social contribution needs. By satisfying these needs, decent work can further promote work fulfillment (Kozan et al., 2019) and mental health (Duffy et al., 2019). In addition, when people are engaged in work that can provide them with safe working conditions, sufficient rest time, and medical security, they are more likely to gain physical health (Duffy et al., 2021). Other studies have shown that decent work positively influences work engagement and job satisfaction (Mcllveen et al., 2021), and negatively influences occupational fatigue (Di Fabio et al., 2021) and turnover intention (Wang et al., 2019). Therefore, this study adopted PWT to build a theoretical framework and to explain the relationship between decent work and its outcomes.

### Employee Well-Being

As an important part of positive psychology, well-being has long been widely concerned by the academic community (Singh et al., 2016). In the early stage of the study, scholars defined well-being only as psychological and health state at the work level, and measured employee well-being from four aspects including job satisfaction, job burnout, emotional exhaustion, and work pressure (Wright and Cropanzano, 2004; Kausto et al., 2005). Subsequently, some scholars proposed that the definition of well-being should not only focus on the state at work, but also pay attention to the psychological feelings based on non-work aspects. For example, Lu et al. (2006) divided well-being into job satisfaction, family satisfaction, life satisfaction and positive emotions. From the perspective of integration, Zheng et al. (2015) proposed that well-being not only reflects employees’ satisfaction with work and life, but also reflects employees’ psychological satisfaction, thus it can be divided into three dimensions: life well-being, workplace well-being and psychological well-being. In addition, in view of the lack of measurement tools in previous studies, Zheng et al. (2015) developed an effective measurement tool on the basis of qualitative and quantitative research, thus making up for the deficiency of existing studies. Therefore, the operational definition of employee well-being by Zheng et al. (2015) was used to carry out the present study.

### Decent Work, Need Satisfaction, and Well-Being

According to PWT, decent work can directly influence individuals’ well-being, and indirectly allow individuals to achieve well-being by satisfying their needs for survival, social contribution, and self-determination (Duffy et al., 2016). Empirical studies show that decent work not only has a positive impact on employees’ job satisfaction and life satisfaction (Chen et al., 2020), it can also make employees perceive the meaning of work (Allan et al., 2019). It can be seen that the three types of need satisfaction fulfilled by decent work are consistent with the three dimensions of employee well-being. Specifically, decent work can meet the survival needs of employees by offering sufficient remuneration for food, shelter, medical care, and other resources, which will in turn promote their life well-being (Blustein et al., 2016). In addition, decent work is also an important avenue for employees to establish social ties with colleagues in the workplace and make contributions to others, which will in turn increase their workplace well-being (Autin et al., 2019). Finally, according to PWT, decent work can fulfill employees’ self-determination needs such as autonomy, competence, and relatedness (Duffy et al., 2016) and the satisfaction of these psychological needs can help improve the psychological well-being of employees (Allan et al., 2016; Van den Broeck et al., 2016). Hence, we propose the following hypotheses:

**Hypothesis 1 (H1):** Decent work has a positive impact on well-being of millennial employees.

**Hypothesis 2 (H2):** Survival need satisfaction mediates the relationship between decent work and well-being of millennial employees.

**Hypothesis 3 (H3):** Social contribution need satisfaction mediates the relationship between decent work and well-being of millennial employees.

**Hypothesis 4 (H4):** Self-determination need satisfaction mediates the relationship between decent work and well-being of millennial employees.

### Comparison of Mediating Effects of Survival Contribution and Self-Determination Need Satisfaction

Self-determination theory holds that individuals have needs for autonomy, competence, and relatedness and tend to interact with the environment in a self-determined way (Deci and Ryan, 2000). Studies have pointed out that millennials are keen on engaging work in which they can exercise autonomy and doing tasks that are conducive to their growth and development (Shri, 2011). This is especially true in the case of Chinese millennials (Lee et al., 2017). Growing up in the era of China’ reform and opening up as well as the rapid development of market economy, the physiological and security needs of millennial employees have been greatly satisfied since their childhood (Zhao, 2018). Therefore, they are more eager to satisfy higher levels of needs such as autonomy, competence and relatedness. Specifically, they not only have a stronger sense of professional achievement, but also yearn for a loose and free working environment where they can have a say in their work (Warner and Zhu, 2018). Additionally, they are eager to have good interpersonal relationships with colleagues in the workplace (Hou et al., 2014).

In sum, many Chinese millennial employees have a strong desire for determining their own life (Ren et al., 2011). Meanwhile, an empirical study by Baard et al. (2004) showed that employees who experienced greater satisfaction of the needs for autonomy, competence, and relatedness at work displayed greater levels of well-being than those who are less satisfied with these needs. Hence, we propose the following hypothesis:

**Hypothesis 5 (H5):** Compared with survival need satisfaction and social contribution need satisfaction, the mediating effect of self-determination need satisfaction is more significant in the relationship between decent work and well-being of millennial employees.

**FIGURE 1:**
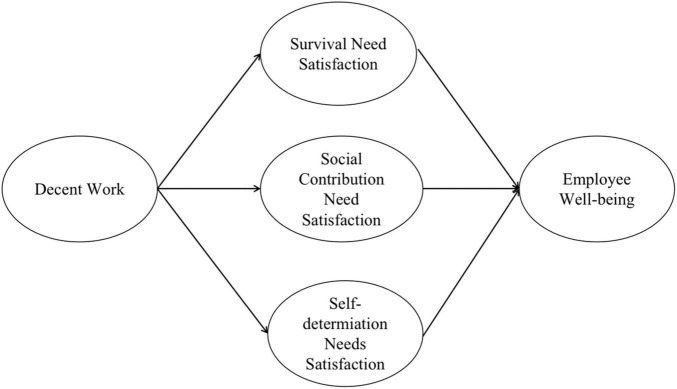
Research model.

The research model reflecting these hypotheses is presented in Figure 1.

## Materials and Methods

### Procedure and Participants

The surveys were carried out from July, 2021 to August, 2021. Data for the present study were collected through the WJX online data collection service,^1^ which is a reliable and popular online tool for academic institutes in China. More specifically, a link to the online questionnaire was disseminated *via* WeChat which is a social media and messaging platform used widely in China. Before taking the survey, participants were informed that working adults born between 1980 and 1999 could successfully submit the questionnaire online and that the survey was related to their experiences with work. The target respondents were informed that the questionnaire would be kept confidential and the results of the survey would only be used for academic purpose. In order to ensure the accuracy of the data, this study excluded those participants who spent less than 2 min to finish our questionnaire, and those who failed the attention check items. Additionally, respondents who did not meet the age criteria were also removed. The final sample consisted of 421 participants with estimated response rate of 85%.

In terms of sample structure, subjects mainly come from the southern part of China, and are distributed in various industries, ranging from the internet, education to manufacturing. Among them, 51.3% are male and 48.7% are female. Married employees account for 48.9% while unmarried employees represent 49.2%. Most employees are between 25 and 29 years old, accounting for 36.8%, followed by those aged between 30 and 34, accounting for 26.1%. In terms of educational level, 20.2% have gained a college degree or less, 58.9% have received a bachelor’s degree and 20.9% hold a master’s degree or above. Regarding tenure, 39.5% of respondents have worked for 3 years or below, 48.3% for 4–9 years, and 24.7% for over 10 years. Most employees worked in private enterprises (48.2%), followed by those working state-owned enterprises (24.9%). The detailed descriptive data of the sample are provided in Table 1.

**TABLE 1:** Demographics characteristics of sample.

Characteristics		Number	Percentage (%)
Gender	Male	216	51.3
	Female	205	48.7
Age	18–24	81	19.2
	25–29	155	36.8
	30–34	110	26.1
	35–39	41	9.7
	>40	34	8.1
Marital status	Unmarried	207	49.2
	Married	206	48.9
	Others	8	1.9
Educational level	College degree or less	85	20.2
	Bachelor’s degree	248	58.9
	Master’s degree	85	20.2
	Doctor’s degree	3	0.7
Tenure	Less than a year	47	11.2
	1–3 years	119	28.3
	4–6 years	84	20.0
	7–9 years	67	15.9
	10 years or more	104	24.7
Nature of enterprise	State-owned enterprises	105	24.9
	Private enterprises	203	48.2
	Foreign enterprises	9	2.1
	Joint ventures	9	2.1
	Government institution	40	9.5
	Others	55	13.1
Total		421	100.0

### Instruments

The scales used in the current study were taken from articles published in internationally renowned journals. The English scale followed the back-translation procedure (Brislin, 1986) and was perfected by a doctor of management with good English. After a small range of filling tests, a formal questionnaire was formed. 7-point Likert scale was used for all items in the questionnaire (1 = “strongly disagree,” 7 = “strongly agree”).

#### Decent Work

Participants’ perceptions of their decent work were measured by the Decent Work Scale, which consists of 15 items assessing five subscales of decent work (i.e., safe working conditions, access to healthcare, adequate compensation, free time and rest, and complementary values) (Duffy et al., 2017). A sample item from each subscale is “I feel emotionally safe interacting with people at work,” “I get good healthcare benefits from my job,” “I am rewarded adequately for my work,” “I have free time during the work week,” and “The values of my organization match my family values.” The Cronbach’s alpha for this scale was 0.921 in the current study.

#### Need Satisfaction

To measure need satisfaction, we used the 20-item Work Need Satisfaction Scale (Autin et al., 2019). The scale includes five subscales that measure the following: survival needs, social contribution needs, competence needs, relatedness needs, and autonomy needs. Participants were presented with the statement, “My work allows me to:” followed by 20 items. Typical items, one from each respective subscale, include the following: “Have the resources to provide nutritious food for myself and my family,” “Make a contribution to the greater social good,” “Feel like I am good at my job,” “Feel like I fit in,” and “Do tasks the way I want.” The Cronbach’s alpha for the five needs subscales in the current study were as follows: survival (0.920), social contribution (0.917), competence (0.852), relatedness (0.875), and autonomy (0.884).

#### Employee Well-Being

Participants’ perceptions of well-being were measured by the Employee Well-Being Scale, which consists of 18 items assessing three subscales (i.e., life well-being, workplace well-being, and psychological well-being) (Zheng et al., 2015). A sample item from each subscale is “I feel satisfied with my life,” “In general, I feel fairly satisfied with my present job,” and “I generally feel good about myself, and I’m confident.” The Cronbach’s alpha for this scale in the current study is 0.906.

### Data Analysis

SPSS 21.0 was used to implement common method bias testing, descriptive analysis and correlation analysis, while AMOS 21.0 was used for confirmatory factor analysis, structural equation model analysis and multiple mediating effect test. Since the variables used in the research model are high-order latent variables, structural equation model can measure the relationship of variables more accurately so that the results of measurement can be closer to the actual results. More specifically, this study first adopted confirmatory factor analysis to access reliability and validity of the measurement model, and then used χ2/*df*, RMSEA, NFI, TLI, CFI, GFI, and AGFI to assess the model-data fit (Hu and Bentler, 1999; Kline, 2015). With regard to the structural model test, the direct effect was examined by path analysis. Moreover, the bootstrap method was used to test the multiple mediation effect.

## Results

### Measurement Model Testing

Since all the items measuring the core variables were self-reported, there might be common method bias. According to suggestions of Podsakoff et al. (2003), Harman single-factor test was conducted to detect common method bias. All the items in the questionnaire were put into an exploratory factor analysis, and the results showed that the first precipitated factor accounted for only 22% of the total variation, which was much lower than the critical value of 40%. Therefore, there was no serious common method bias in the data obtained in this study.

In addition, factor loading, composite reliability (CR) and average variance extracted (AVE) were used to assess the reliability and validity concerns. Specifically, internal consistency reliability can be measured by CR which should be higher than 0.7. If the value of both factor loading and AVE is higher than 0.5, then the data can show good convergent validity (Fornell and Larcker, 1981). Table 2 illustrates the reliability and convergent validity of our data, and all critical values are met. Besides, Table 3 shows that all the square root of AVE are larger than the correlations of each construct, which further suggests good discriminant validity.

**TABLE 2:** Reliability and convergent validity.

Variable	Indicator	No. of items	Factor loading	CR	AVE
DW	Safe working conditions	3	0.891–0.926	0.931	0.818
	Access to healthcare	3	0.783–0.846	0.804	0.654
	Adequate compensation	3	0.825–0.838	0.872	0.695
	Free time and rest	3	0.843–0.913	0.909	0.769
	Complementary values	3	0.844–0.877	0.897	0.743
SN	Survival needs	4	0.833–0.917	0.921	0.745
SCN	Social contribution needs	4	0.842–0.880	0.917	0.735
SDN	Autonomy needs	4	0.723–0.806	0.852	0.591
	Competence needs	4	0.746–0.833	0.876	0.638
	Relatedness needs	4	0.767–0.856	0.885	0.658
WE	Workplace well-being	6	0.769–0.894	0.922	0.663
	Life well-being	6	0.750–0.883	0.921	0.662
	Psychological well-being	6	0.730–0.842	0.912	0.634

*DW, decent work; SN, survival need satisfaction; SCN, social contribution need satisfaction; SDN, self-determination need satisfaction; EW, employee well-being; CR, composite reliability; AVE, average variance extracted.*

**TABLE 3:** Correlation coefficient matrix and discriminant validity.

Variable	Mean	SD	1	2	3	4	5
1. DW	4.657	1.024	**0.858**				
2. SN	4.898	1.333	0.153**	**0.863**			
3. SCN	5.303	1.183	0.138**	0.178**	**0.857**		
4. SDN	4.987	0.958	0.172**	0.085	0.264**	**0.793**	
5. EW	4.956	0.905	0.263**	0.156**	0.472**	0.500**	**0.808**

N = 421; **p < 0.01 (two-tailed tests); DW, decent work; SN, survival need satisfaction; SCN, social contribution need satisfaction; SDN, self-determination need satisfaction; EW, employee well-being.

*Figures in bold on diagonals are the square root of AVE of each construct.*

The correlations for the four variables of this study are also reported in Table 3. Decent work was positively related to survival need satisfaction (*r* = 0.153, *p* < 0.01), social contribution need satisfaction (*r* = 0.138, *p* < 0.01), self-determination need satisfaction (*r* = 0.172, *p* < 0.01), and employee well-being (*r* = 0.263, *p* < 0.01) separately. Besides, employee well-being was positively related to survival need satisfaction (*r* = 0.156, *p* < 0.01), social contribution need satisfaction (*r* = 0.472, *p* < 0.01) and self-determination need satisfaction (*r* = 0.500, *p* < 0.01), respectively. Thus, the correlations showed preliminary support for our hypotheses.

### Structural Model Testing

**FIGURE 2:**
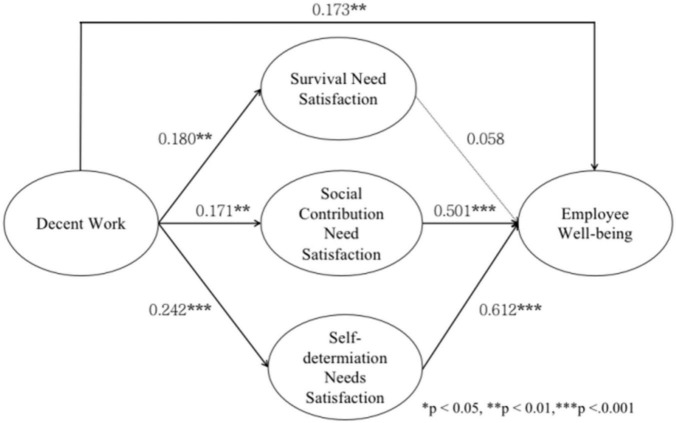
Results of path analysis. *p < 0.05, **p < 0.01, ***p < 0.001.

This study tested a structural equation model that contained all the variables of this study. This model had good fit to the actual data: χ2/*df* = 2.497, RMSEA = 0.060, NFI = 0.911, TLI = 0.935, CFI = 0.945, GFI = 0.920, and AGFI = 0.895. All fit indexes of the model reached the satisfactory level, so we further analyzed the path of the model. According to the results of path analysis shown in Figure 2, decent work had a significant positive impact on employee well-being (β = 0.173, *p* < 0.01). Therefore, hypothesis 1 was supported.

In order to investigate the significance of the mediation effects, this study ran the bootstrap method in AMOS21.0, selected 5000 repetitions, set the confidence interval standard as 95% according to the recommendations of Shrout and Bolger (2002). If the confidence interval does not include 0, the specific mediating effect is significant; if the confidence interval includes 0, the specific mediating effect is insignificant (Hillis and Bull, 1993; Cheung and Lau, 2008). Moreover, since this research model is a multiple mediation model, a separate analysis can only get the result of overall mediation effect rather than the specific mediation effect. Therefore, this study used the syntax of AMOS to assign values to all the relevant paths, and calculated the specific mediation effect of non-standardization and standardization, respectively.

As shown in Table 4, decent work was found to have a significant, indirect effect on employee well-being *via* the satisfaction of both social contribution needs [estimate effect = 0.086, 95% confidence interval = (0.019, 0.155)] and self-determination needs [estimate effect = 0.151, 95% confidence interval = (0.047, 0.259)]. However, the indirect effect of decent work on employee well-being *via* the satisfaction of survival needs was insignificant [estimate effect = 0.010, 95% confidence interval = (−0.012, 0.044)]. Thus, hypothesis 3 and hypothesis 4 were supported while hypothesis 2 was not supported.

**TABLE 4:** Bootstrap analysis of mediating effect test.

Hypothesized path		Estimate	Lower	Upper	*P*-Value
H2	DW → SN → EW	0.010	−0.012	0.044	0.404
H3	DW → SCN → EW	0.086	0.019	0.155	0.017
H4	DW → SDN → EW	0.151	0.047	0.259	0.007

*DW, decent work; SN, survival need satisfaction; SCN, social contribution need satisfaction; SDN, self-determination need satisfaction; EW, employee well-being.*

After identifying the mediating effects of social contribution need satisfaction and self-determination need satisfaction, this study also compared the significance of the mediating effects. As shown in Table 5, self-determination need satisfaction played a stronger mediating effect between decent work and employee well-being compared with social contribution need satisfaction. Thus, hypothesis 5 was supported.

**TABLE 5:** The comparison of mediating effects.

Hypothesized path	c	a * b	c”	a * b/c

	Total effect	Indirect effect	Direct effect	
DW → SCN → EW	0.258	0.086	0.198	32.9%
DW → SDN → EW	0.323	0.151	0.198	46.4%

*DW, decent work; SCN, social contribution need satisfaction; SDN, self-determination need satisfaction; EW, employee well-being.*

## Discussion

Employee well-being can not only reduce absenteeism rate and turnover rate, but also stimulate individual potential and improve organizational performance (Gillet et al., 2012). Therefore, investigating what contributes to employees’ sense of happiness is of great significance to academia and business. Within a PWT framework, the current study is the first to examine how and why decent work might relate to well-being of millennial employees.

After analyzing data from 421 millennial employees in China, this study found that decent work had a positive impact on well-being of millennial employees. Social contribution need satisfaction and self-determination need satisfaction partially mediated the positive relationship between decent work and well-being of millennial employees, but the mediating role of survival need satisfaction was not significant. Compared with social contribution need satisfaction, self-determination need satisfaction had a more significant mediating effect on well-being of millennial employees.

The findings of this study should be comprehended by taking cultural contextual factors into consideration. The current Chinese millennials grew up in an era of rapid economic development and have been greatly satisfied with their material life (Zhao, 2018). Compared with older generations, millennial employees are no longer satisfied with survival needs, which could probably explain why the satisfaction of survival needs does not bring them feelings of happiness. Moreover, Chinese millennial employees are more in favor of autonomy and control, and they are in the learning phase and eager to improve their working capacity (Fang et al., 2020). Therefore, it is understandable that self-determination need satisfaction plays a stronger mediating effect between decent work and well-being of millennial employees.

## Theoretical Contributions

The current study has three theoretical implications. First, the current study was the first to examine how the experience of decent work relates to well-being of millennial employees in the Chinese context. Mirroring previous studies (Kozan et al., 2019), decent work can positively influence employee well-being. However, unlike previous studies on well-being which just focused on job satisfaction and life satisfaction (Kozan et al., 2019), this study integrated life well-being, workplace well-being and psychological well-being and provided empirical evidence to verify that decent work could bring overall happiness to Chinese millennial employees. Therefore, this study enriches the research on the outcome variables of decent work.

Second, responding to the call of Duffy et al. (2019) that future studies should examine how decent work impacts well-being of individuals *via* need satisfaction, this study explored need satisfaction as an underlying mechanism in this relationship. In line with some propositions within PWT (Duffy et al., 2016), this study found that social contribution need satisfaction and self-determination need satisfaction partially mediated the relationship between decent work and well-being of millennial employees although the mediating effect of survival need satisfaction was found to be insignificant. Thus, this study enriches the research on the antecedent variables of well-being of millennial employees.

Finally, the current study expands the scope of the application of PWT given the fact that previous studies have been conducted almost exclusively in Western, individualist cultural context (Allan et al., 2019; Mcllveen et al., 2021). Since Duffy et al. (2016) proposed that decent work can promote work fulfillment and well-being by satisfying individuals’ survival needs, social contribution needs and self-determination needs, a number of empirical studies have been conducted to examine the relations between decent work and well-being outcomes. For example, Duffy et al. (2019) found that securing decent work can promote increased mental health primarily because work is meeting individual needs and may promote physical health by meeting survival needs. Furthermore, Duffy et al. (2021) found that decent work can predict heath behaviors indirectly *via* survival need satisfaction. However, no studies to date have examined whether there are differences in the mediating effects of survival, social contribution and self-determination need satisfaction. By comparing the significance of the mediating effect, this study found that the mediating effect of self-determination need satisfaction was more significant between decent work and well-being of millennial employees than that of social contribution need satisfaction. Therefore, this study extends the application of PWT in a non-western, collectivist cultural context.

## Practical Implications

The results from this study have several implications for HR management and practices. First, managers should motivate millennial employees from the work itself. In other words, it is necessary for managers to provide decent work for millennial employees to enhance their well-being. To achieve this goal, managers could design staff’s work content, working hours, salary, and welfare in a scientific and reasonable manner, and create a free and comfortable working environment.

Second, when managing millennial employees, organizations should not blindly apply traditional management methods, but should strive to meet their psychological needs according to their values and behavior preferences. For instance, increasing autonomy levels, promoting skills development, and strengthening staff relationship can be conducive to meeting millennial employees’ needs for self-determination, thereby enhancing their well-being.

Third, given that millennial employees pay more attention to self-determination needs, managers could delegate effectively and empower them to carry out their roles with adequate resources. Besides, implementing flexible working system can also encourage employees to decide their own working hours and even where they work as long as they can finish their tasks before deadlines. This can not only satisfy the autonomy needs of millennial employees (Westerman and Yamamura, 2007), but also enables them to achieve work-life balance (Twenge, 2010).

Finally, managers should make an effort to create a free communication atmosphere to understand the needs and expectations of millennial employees. It is important for managers to communicate with their younger staff in a respectful, caring and equal manner, to give them the opportunity to express their opinions, and to encourage them to provide valuable suggestions for the development of organizations.

## Limitations and Directions for Future Research

This study also has some limitations to be solved in the near future. Firstly, all the questionnaires collected in this study were self-reported by employees, so this single source of information may lead to some deviation in variable measurement. Future research could collect questionnaires in the form of joint reports by supervisors and employees so as to obtain more accurate data. Secondly, considering the length of the article, this study did not independently propose the relationship between each dimension of decent work and other variables. It is advisable that future research should divide decent work into several dimensions and explore the relationship between each dimension and employee well-being. Thirdly, this study only focused on work factors that lead to employee well-being, so we did not explore leadership styles which is equally important in inducing well-being (Ding and Yu, 2021). It is also necessary to consider the boundary conditions of leadership style in the relationship between decent work and employee well-being. Finally, due to the cross-sectional design, the causality between variables cannot be guaranteed. Future research could make up for this by collecting longitudinal data to make the results more persuasive.

## Data Availability Statement

The original contributions presented in the study are included in the article/supplementary material, further inquiries can be directed to the corresponding author.

## Author Contributions

WW: conceptualization, formal analysis, writing original draft, and writing and editing. WW and TC: investigation. Both authors have read and agreed to the published version of the manuscript.

## Conflict of Interest

The authors declare that the research was conducted in the absence of any commercial or financial relationships that could be construed as a potential conflict of interest.

## Publisher’s Note

All claims expressed in this article are solely those of the authors and do not necessarily represent those of their affiliated organizations, or those of the publisher, the editors and the reviewers. Any product that may be evaluated in this article, or claim that may be made by its manufacturer, is not guaranteed or endorsed by the publisher.

## Footnotes

1
www.wjx.cn
